# Danger in the Classroom: Elemental Mercury Poisoning in Primary School Students

**DOI:** 10.5152/eurasianjmed.2025.251062

**Published:** 2025-11-25

**Authors:** Talip Vural, Ahmet Nezih Kök, Beytullah Ural, Büşra Baydemir Kılınç

**Affiliations:** 1 Department of Forensic Medicine, Atatürk University Faculty of Medicine, Erzurum, Türkiye; 2 Department of Morgue, Council of Forensic Medicine, Erzurum, Türkiye

**Keywords:** Forensic, medicine, mercury poisoning, pediatrics, toxicology

## Abstract

**Background:**

Mercury is a heavy metal that has the potential to exert a deleterious effect on human health. The purpose of this article is threefold: firstly, to raise awareness of the issue; secondly, to offer solutions; and thirdly, to contribute to the existing literature on the subject by examining cases of elemental mercury poisoning that occurred in a primary school.

**Methods:**

In 2019, 34 cases of mercury poisoning due to mercury bottle (used in the laboratory) spillage in a primary school were examined, and control examinations were performed in 2024.

**Results:**

The demographic composition of the cases was as follows: 58.8% of the subjects were male, with an average age of 9 years. The research revealed that 47.1% of cases were exposed to elemental mercury for a period of 4 days, 76.5% of cases involved manual contact with mercury, 82.4% of cases presented symptoms, the mean blood mercury level was 46.62 μg/L, and 2,3-dimercaptopropane-1-sulfonic acid (DMPS) treatment was administered to the cases.

**Conclusion:**

Elevated blood mercury levels were found to be associated with more severe clinical manifestations. The study concluded that a 7-day intravenous administration of DMPS exhibited a high degree of therapeutic efficacy. In order to prevent poisoning from mercury and similar substances in educational establishments, it is essential to raise awareness of toxic substances, establish emergency chemical safety and medical health protocols, and prohibit the use of toxic substances such as mercury and devices containing these substances.

**Main Points**

- Elemental mercury-related mass poisonings among students are still observed in primary schools today.
- Prolonged exposure to mercury is associated with elevated blood mercury levels and the progression of more severe clinical manifestations.
- A 7-day intravenous administration of 2,3-dimercapto-1-propanesulfonic acid has proven to be a highly effective treatment.
- Emergency chemical safety and medical health protocols should be established in schools, and the use of toxic substances such as mercury, as well as devices containing these substances, should be prohibited.

## Introduction

Mercury (Hg) is a heavy metal that occurs naturally in the environment, including air, water, and soil. It has the potential to cause serious toxic effects on human health through environmental and occupational exposure. Elemental (metallic) mercury, organic mercury (methyl and ethylmercury), and inorganic mercury (mercuric chloride) are all found in the environment. These forms have been shown to have serious toxic effects.^1^^-^^4^ Elemental mercury is a shiny, silvery, odorless, and easily evaporated substance. The elemental form of mercury is found in instruments such as sphygmomanometers, thermometers, barometers, batteries, electrical appliances, and in laboratories. Exposure to elemental mercury typically occurs in instances where metallic mercury is spilled or where products containing metallic mercury are broken, resulting in the mercury coming into contact with the atmosphere. Exposed mercury evaporates easily at room temperature, is taken into the body by inhalation, is lipid-soluble, and binds to the proteins of enzymes in the body, producing toxic effects.^3^^-^^5^ The clinical manifestations of mercury intoxication are contingent upon the specific form of mercury, the route, and duration of exposure. It is widely acknowledged that all forms of mercury have different toxic effects on the nervous, digestive, and immune systems, as well as the lungs, kidneys, skin, and eyes. This has led the World Health Organization to designate mercury as one of the 10 most important chemicals for public health.^1^^,^^2^^,^^5^

Elemental mercury poisoning in a primary school setting poses a grave threat to the health of children. It is well established that children are significantly more sensitive to chemicals than adults. Consequently, exposure to toxic substances such as mercury can result in irreversible damage to numerous physiological systems. The issue of mercury exposure in areas where children congregate, such as schools, is a situation that requires urgent intervention. This is not only of concern for individual health but also for public health.^1^^,^^4^^,^^6^

In this article, a case study of 34 cases of elemental mercury poisoning in a primary school population is analyzed. The subsequent development of the incident, the potential health risks involved, the intervention process, and the possible prevention strategies were evaluated in the context of the extant literature. The objective of the study was 2-fold: firstly, to raise awareness of the incident; and secondly, to contribute to the existing literature on the subject by shedding light on the gravity of the incident.

## Materials and Methods

### Case Selection, Data Collection, and Groups

In September 2019, 34 cases of mercury poisoning were reported in a primary school (eighth grade). The cases were attributed to a mercury bottle (utilized in a laboratory setting) falling to the ground and breaking, resulting in the spill of mercury. The affected individuals subsequently applied to Atatürk University in November 2024 for the preparation of a forensic report. Forensic documents issued to the cases following the incident (2019), crime scene investigation documents, sociodemographic data, duration of interaction with mercury and route of transmission, blood mercury levels, medical complaints and clinical findings of the cases, hospitalization and treatment processes were examined in detail. Ethical committee approval was received from the Ethics Committee of Medical Faculty of Atatürk University (Decision no: 5, Meeting no: 1, Date: 31.01.2025). Written informed consent was obtained from each of the cases and their relatives, and detailed clinical examinations were performed with the participation of pediatric clinical specialists in November-December 2024. Blood and urine samples were collected from the patients. Routine blood and urine tests were performed. Liver and kidney function were examined. Blood tests were performed and blood mercury levels were analyzed. The data of the cases at the time of the event (2019) and 5 years after the event (2024) were compared. The study yielded statistically significant findings, which were emphasized.

### Statistical Analysis

The IBM SPSS 25 (IBM SPSS Corp.; Armonk, NY, USA) statistical program was utilized for the statistical analysis of this study. The Kolmogorov–Smirnov test was employed to ascertain the normality of continuous variables (*P* > .05). The independent samples *t*-test was employed to compare the means of 2 groups for normally distributed data, while the one-way ANOVA was utilized to compare the means of 3 or more groups for normally distributed data. *P-*values of less than .05 were considered to be statistically significant. The existence of a relationship between the variables was statistically confirmed.

## Results

### Socio-Demographic Data and Crime Scene Investigation Findings

The demographic composition of the cases was as follows: 58.8% (n = 20) of the cases were male, 41.2% (n = 14) were female, and the mean age was 9 years (min = 6; max = 14; SD = 2.9). Of the cases, 39.4% (n = 10) were students in the class where the mercury was spilled (eighth grade), while 70.4% (n = 24) were students in neighboring classes (Tables 1 and 2). Following the spillage of elemental mercury in the primary classroom, it was ascertained that the substance had been transported to other classrooms by students from the initial classroom or by students from other classrooms. The presence of the elemental mercury in the primary school was observed to last approximately 4 days. During this period, the substance was utilized as a recreational material by the students. However, it was only when clinical symptoms began to manifest that the authorities became aware of the issue. Consequently, the entire school was subjected to a thorough cleaning process, and the students were transported to medical facilities for comprehensive health assessments.

### Duration of Interaction, Route of Transmission, and Clinical Findings

The duration of interaction with elemental mercury was found to vary between 1 and 4 days, with 47.1% (n = 16) of cases exposed to elemental mercury for 4 days. The study revealed that 76.5% (n = 26) of the cases involved direct contact of the mercury by hand, while 73.5% (n = 25) of the cases involved close olfactory exposure to the mercury, with the substance being brought into close proximity with the nose. The study revealed that 82.4% (n = 28) of the cases exhibited clinical findings and medical complaints, while 17.6% (n = 6) did not manifest any medical complaints or clinical findings. However, elemental mercury was detected in the blood sample collected during the health check. The prevalence of gastrointestinal symptoms was found to be 61.8% (n = 21), neurological symptoms 35.3% (n = 12), and skin symptoms 32.4% (n = 11) (Table 2).

### Blood Mercury Level and Treatment Process After the Incident

The mean blood mercury level of the cases following the incident was 46.62 μg/L (min = 11.68; max = 264.9; SD = 62.11) (Table 1). The study revealed that 20.6% (n = 7) of the patients were admitted to the intensive care clinic, while 79.4% (n = 27) were admitted to the pediatric clinic. 2,3-dimercaptopropane-1-sulfonic acid treatment was administered for 7 days in 20.6% (n = 7) and 5 days in 79.4% (n = 27) of the cases (Tables 2 and 3).

### Blood Mercury Levels and Results 5 Years After the Event

**Figure 1.**
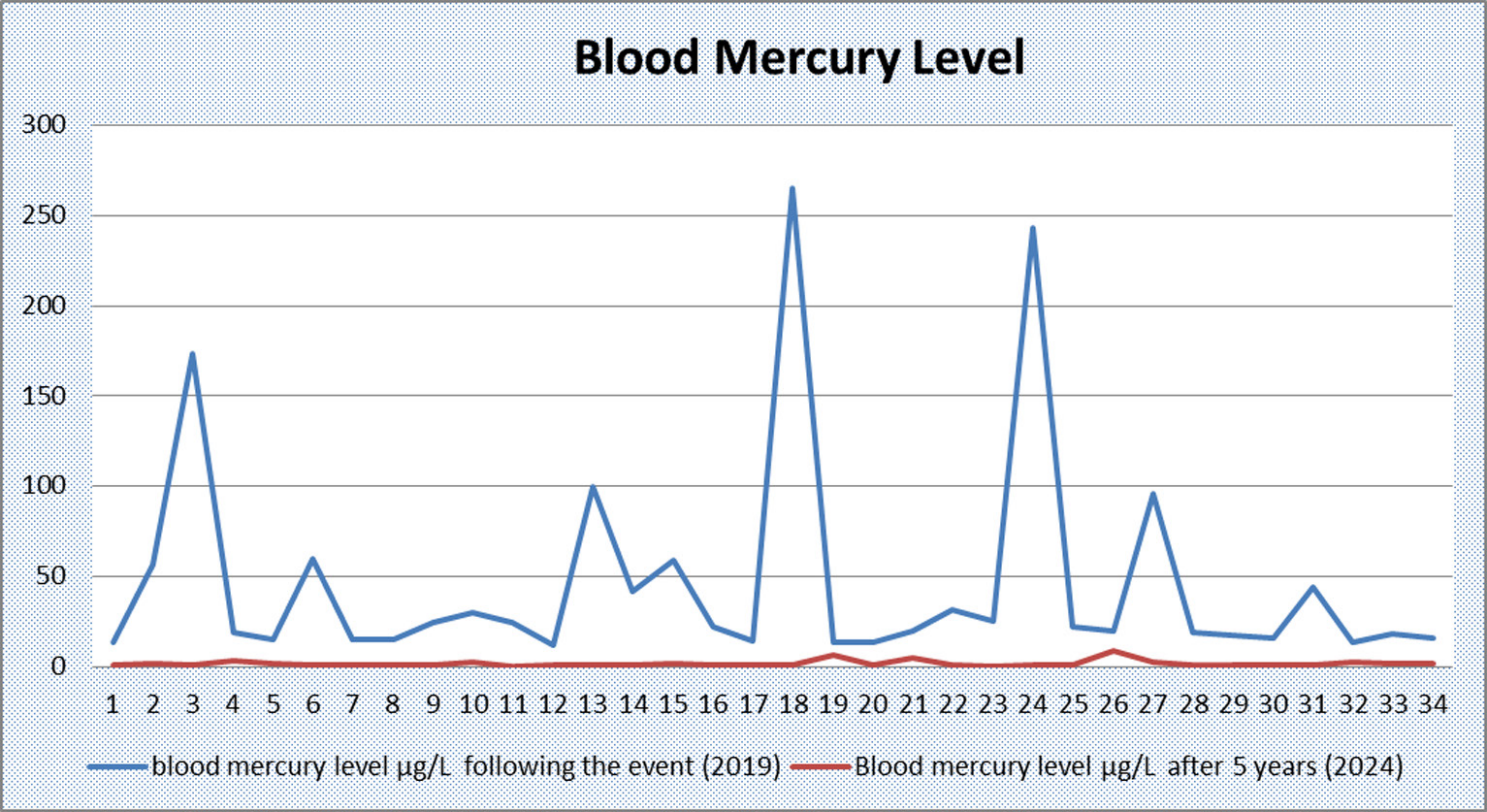
Blood mercury detected after the incident (2019) and 5 years after the incident (2024).

The mean blood mercury level of the cases 5 years after the event was 1.74 μg/L (min = 0.41; max = 8.98; SD = 1.73). Further analysis revealed that the glomerular filtration rate and liver function tests were within the normal range. No clinical findings were detected in the cases. A complete blood count analysis was conducted, revealing microcytosis and hypochromia in 32.4% (n = 11) of the cases. Proteinuria was detected in 14.7% (n = 5) of patients in complete urine analysis (Table 1). After 5 days of treatment, the mean mercury level was 1.89 μg/L and after 7 days of treatment, the mean mercury level was 0.83 μg/L (Table 3) (Figure 1).

### Statistically Significant Findings

The findings of the study indicated a statistically significant increase in blood mercury levels in cases where proximity to the mercury source was observed (i.e., the classroom where mercury had been spilled) (*P* = .021). Furthermore, an increased duration of interaction with mercury was also identified (*P* = .001), as was close interaction with mercury and hand contact (*P* = .04). Statistically significant associations were identified between elevated blood mercury levels and clinical and medical complaints (*P* = .018) and neurological findings (*P* = .04). Blood mercury levels were found to be statistically higher in patients who were hospitalized in the intensive care unit (*P* = .001) and who received 7-day treatment (*P* = .001) (Table 2). The mean blood mercury levels 5 years later were statistically lower (*P* = .004) in patients treated for 7 days compared to patients treated for 5 days (Table 3).

## Discussion

Mercury is considered to be one of the most toxic elements for human health, representing a significant public health concern. It is evident that the propensity of mercury to vaporize, coupled with its rapid absorption through inhalation, can pose significant health hazards in public living areas, such as educational institutions.^1^^,^^2^^,^^5^ The present study is an evaluation of poisoning cases due to elemental mercury exposure in a primary school. The findings reveal once again how vulnerable children are to toxic substances such as mercury in enclosed spaces.

It is widely acknowledged that children are more vulnerable to the effects of toxins, such as mercury, than adults. This heightened susceptibility can be attributed to a number of factors, including differences in metabolism, behavioral patterns, growth and changes in organ systems and functions, and sensitivity to environmental toxins.^2^^,^^4^^,^^7^ In the event of mercury being released into the environment as a consequence of a broken bottle, even in minute quantities, the substance has the potential to evaporate rapidly, a phenomenon that is especially pertinent in enclosed spaces such as classrooms. This has the potential to have more severe consequences for children, given their higher respiratory rate when compared to adults.^6^^-^^8^ The mean age of the cases under consideration was 9 years (min = 6; max = 14), and the incident occurred in a closed classroom environment. As indicated in the extant literature, the location of the incident and the age demographics of the affected groups are of particular concern. Consequently, mercury-containing materials (e.g., mercury thermometers, barometers, fluorescent lamps, mercury-based laboratory chemicals) should be completely eliminated from educational institutions. It is recommended that these be replaced with digital and mercury-free alternatives.

Repeated exposure to low doses of mercury, cumulative exposure, or inhalation of large amounts can cause serious toxic effects. As the duration of interaction with elemental mercury increases, the severity of damage increases.^4^^,^^6^^,^^7^ In the present study, the duration of interaction with mercury was found to vary between 1 and 4 days. A statistically significant increase in blood mercury levels was observed as the duration of interaction with mercury increased. A further significant issue pertains to the cleaning of mercury that has been spilled into the environment. It is imperative to note that the use of brooms, vacuum cleaners, and vacuuming is strictly prohibited when it comes to the cleaning of elemental mercury. In such cases, there is a risk of significant releases of mercury vapor into the environment, which can result in new instances of poisoning.^6^^,^^7^^,^^9^ In the present case, in addition to the students, 2 personnel responsible for cleaning the mercury were poisoned by mercury. Consequently, in such unfavorable circumstances, cleaning should be expeditiously executed, meticulously, and with the assistance of health services by a professional team. It is imperative to acknowledge that inadequate cleaning practices can lead to an elevated risk of poisoning.

Elemental mercury is distinguished by its unique property of being the only metal that exists as a liquid at room temperature. This characteristic, coupled with its high vapor pressure, renders it susceptible to easy release into the atmosphere in the form of mercury vapor. Human exposure to elemental mercury occurs primarily via inhalation, resulting in rapid absorption and distribution to all major organs. Furthermore, it can be absorbed through the gastrointestinal tract or by smell from the nasal cavity.^3^^,^^5^^,^^10^ The present study set out to investigate the relationship between hand-to-mouth contact with mercury and the subsequent impact on blood mercury levels in students. The study found that 76.5% of students had touched mercury by hand for play purposes when mercury in a closed bottle was spilled on the floor in the classroom. The study concluded that blood mercury levels of students who touched mercury by hand were statistically significantly higher than those who did not. Despite the existence of studies that suggest dermal absorption of mercury to be limited, it is hypothesized that hand contact with mercury results in more severe toxic manifestations due to the facilitation of both olfactory perception and inhalation.^10^^,^^11^ Furthermore, the dermal absorption of mercury is a subject that requires elucidation through detailed studies.

Elemental mercury is defined as an uncharged monoatomic form that is highly diffusible and lipid-soluble. Elemental mercury has been demonstrated to traverse the blood-brain barrier, the blood-placental barrier, and the lipid bilayers of cellular and intracellular organelle membranes.^4^^,^^5^^,^^10^ Elemental mercury poisoning can result in severe local and systemic clinical presentations, both in acute and chronic phases. In instances of acute exposure, the following symptoms may be observed: weakness, memory loss, metallic taste in the mouth, salivation, mercury stomatitis, dermatosis, vomiting, bloody diarrhea, necrosis of the intestinal mucosa, respiratory tract irritation, fever, cough, dyspnea, chest pain, interstitial pneumonia, bronchiolitis and pulmonary edema, nephrotic syndrome and blood pressure changes, and various skin manifestations such as wounds and rashes. Acute exposure to elevated levels of mercury vapor has been demonstrated to result in severe pulmonary damage, and in extreme cases, death, due to the onset of hypoxia.^1^^,^^2^^,^^10^^-^^14^ In the present study, 82.4% of patients exhibited clinical indications consistent with acute mercury poisoning, as reported in the extant literature. Despite the existence of studies that have reported an absence of correlation between serum mercury levels and the presence of symptoms, as well as clinical severity, the present study has found a statistically significant correlation between clinical severity and serum mercury levels.^15^^,^^16^ Gastrointestinal complaints, including abdominal pain, nausea, and vomiting, were frequently reported. However, neurological symptoms, such as headache, dizziness, and weakness, were found to be statistically significantly higher as the serum mercury level increased. In 17.6% of cases, despite the absence of any symptomatic condition, elevated levels of mercury were detected in blood serum during health screenings. This situation demonstrates that mercury poisoning can, on occasion, be asymptomatic. Consequently, when mercury poisoning is suspected, it is imperative that routine health screening is performed on all individuals in the environment, irrespective of their clinical status. In the present study, it is asserted that it is of paramount importance and value to transport all students and other members of staff at the location of the incident to hospitals via the Ministry of Health, in order to undergo health screenings.

It is evident that in cases of chronic exposure to elemental mercury, the central nervous system and kidneys are the organs most susceptible to the toxic effects of the substance. The clinical manifestations of this condition include erythema, a form of toxic organic psychosis, psychic disorders, memory loss, impairment of sensory and motor conduction systems, gingivitis and tooth loss, proteinuria and nephrotic syndrome, impaired liver function, triggering of autoimmune diseases, impairment of the immune system, and skin disorders.^5^^,^^10^^,^^17^^,^^18^ Furthermore, the potential involvement of inorganic mercury in the development of Alzheimer’s disease has been postulated.^19^ In the present study, liver and kidney function tests, in addition to detailed clinical examinations in the domains of neurology, psychiatry, and pediatrics, were found to be within normal parameters 5 years later. In accordance with the extant literature, proteinuria was identified in 14.7% of cases. However, microcytosis and hypochromia were identified in 32.4% of cases upon complete blood count analysis. This condition has not been documented in the extant literature. No pathological condition was detected in the cases that could explain this phenomenon. Consequently, it was hypothesized that microcytosis and hypochromia may be among the long-term effects of elemental mercury poisoning, in addition to the literature. It was proposed that this situation should be investigated with detailed scientific studies.

In cases of acute elemental mercury poisoning, the concentration of mercury in whole blood is a critical indicator immediately following a short-term exposure of a high magnitude. As a general rule, the concentration of mercury in whole blood is typically less than 10 μg/L; however, it has been observed to increase in proportion to the duration of exposure to mercury vapor. For non-occupational exposure, the standard level is considered to be less than 10 μg/L. Subsequent to exposure, a decline in blood mercury levels is observed, with the concentration of mercury in urine serving as an indicator of body mercury burden. This is considered a superior biomarker of long-term exposure to elemental mercury. Furthermore, hair and nail analysis is utilized to substantiate the presence of mercury or its toxicity. However, it is imperative to acknowledge that these data, in isolation, are inadequate for conclusive determination.^5^^,^^13^^,^^20^^-^^22^ In the present cases, blood mercury levels were evaluated in the acute period following the event, and the mean blood mercury level was found to be 46.62 μg/L (min = 11.68; max = 264.9). Consequently, the evaluations conducted 5 years later encompassed the assessment of blood mercury levels within the parameters of the laboratory’s capabilities. It is acknowledged that the absence of an analysis of urine mercury levels, attributable to economic and laboratory constraints, constitutes a lacuna in the present study.

The primary objective in the management of mercury poisoning is to immediately cease exposure to mercury and to reduce mercury concentrations in critical organs or injured areas. In the event of direct contact with mercury, the affected skin area should be thoroughly cleansed with soap and water. In instances of primary management of acute exposure to elemental mercury vapors in situations of severe toxicity, vital organ monitoring is imperative. The administration of supplemental oxygen, endotracheal intubation, and mechanical ventilation is recommended.^10^^,^^13^^,^^23^ Chelating agents such as penicillamine, Dimercaprol, or British anti-Lewisite, Meso 2,3-dimercaptosuccinic acid (Succimer, DMSA), 2,3-dimercapto-1-propane sulfonic acid (Unithiol, DMPS), mono isoamyl ester of DMSA (MiADMSA), and combination therapy of DMSA and MiADMSA are utilized to ensure mercury excretion. Plasma exchange-hemodialysis-plasmapheresis is initiated approximately 24-36 hours after clinical diagnosis when the patient’s life is in danger and there is no suitable alternative treatment. Plasma exchange is a therapeutic intervention that can be employed in emergency situations where elevated plasma concentrations of pathogenic substances are present.^24^^-^^28^ In accordance with the literature, the treatment was administered intravenously for a period of 7 days to patients hospitalized in the pediatric intensive care unit and for a period of 5 days to patients hospitalized in the pediatric clinic. The treatment in question was DMPS. It is a water-soluble analog of dimercaprol with a chemical formula of C_3_H_7_O_3_S_3_Na. Its utilization has been sanctioned in Russia and other former Soviet countries since 1958, in Germany since 1976, and in the USA since 1999. This has resulted in the replacement of DMSA in Europe. The administration of DMPS can be performed either orally or via intravenous injection. The duration of treatment is contingent upon the mercury concentration in the blood and urine. The substance in question has been shown to penetrate renal cells, thus facilitating the removal of mercury accumulated in renal tissues. In turn, this mercury is then excreted in the urine. Adverse effects are uncommon, but cases of rash, nausea, and leukopenia have been documented.^13^^,^^29^^,^^30^ In addition to the existing literature on the subject, patients frequently complained of a metallic taste in the mouth during treatment. The investigation revealed that a 7-day treatment regimen led to a statistically significant decrease in blood mercury levels in comparison with a 5-day treatment duration. The present study found that the results of DMPS treatment were quite successful. Consequently, it was proposed that DMPS treatment should be prioritized in cases of possible mercury poisoning and that it is more beneficial to routinely extend the duration of hospitalization to 7 days and administer 7-day intravenous treatment.

This study has several limitations. It is a single-center study and consists of rare cases. Since the study involves forensic cases, evaluations were made based on medical documents provided by forensic authorities. For mercury levels, only blood analyses were performed, both in the past 5 years and currently. Due to the non-routine nature and high cost of laboratory procedures, urinary mercury analysis could not be conducted. In the event of a similar incident in the future, if adequate financial resources are secured, mercury levels could be analyzed in both blood and urine samples.

In conclusion, in this study, it was proved that the closer to the elemental mercury source, the longer the time of interaction with mercury and the longer the hand contact with mercury, the higher the blood mercury level, the more severe the clinical picture and the more prominent the neurological symptoms, and that the 7-day intravenous use of DMPS was highly effective in the treatment. Five years after the incident, microcytosis and hypochromia were detected in 32.4% of the cases in a complete blood count analysis, and this situation needs to be examined in detailed scientific studies.

On the other hand, although elemental mercury is a serious toxic element for human health, it is still used in schools today and causes serious mass poisonings in students. Therefore, it is necessary to increase toxic substance awareness in all educational institutions, to establish emergency chemical safety and medical health protocols, and to ban the use of toxic substances such as mercury and devices containing these substances in all schools.

## Figures and Tables

**Table 1.** Comparison of Findings at the Time of Elemental Mercury Exposure and 5 Years Later

Case	Sex	At the Time of Mercury Exposure (2019)				5 Years After the Date of Mercury Exposure (2024)		
		Age	Exposure to Elemental Mercury (Day)	Blood Mercury Level (μg/L)	Duration of Treatment (Day)	Blood Mercury Level (μg/L)	LFTs and GFR	CBC
C1	M	7	3	13.69	5	1.69	NR	NR
C2	F	7	5	56.78	5	0.87	NR	NR
C3	F	13	5	173.28	7	1.12	NR	NR
C4	M	7	2	18.67	5	3.17	NR	NR
C5	F	13	5	14.63	5	1.68	NR	NR
C6	M	14	5	59.68	7	1.01	NR	NR
C7	F	6	3	14.81	5	1.09	NR	NR
C8	M	8	5	15.18	5	1.24	NR	NR
C9	F	8	3	24.54	5	0.83	NR	NR
C10	M	6	5	29.65	5	2.42	NR	MC and HC
C11	F	6	4	24.13	5	0.42	NR	NR
C12	M	9	2	11.68	5	1.23	NR	MC and HC
C13	M	13	5	99.55	7	0.68	NR	NR
C14	F	6	5	41.67	5	1.25	NR	MC and HC
C15	F	13	5	59.23	7	0.86	NR	NR
C16	F	7	2	22.14	5	1.42	NR	NR
C17	F	9	2	14.43	5	1.25	NR	NR
C18	F	13	5	264.9	7	0.87	NR	MC and HC
C19	M	7	2	13.43	5	6.21	NR	MC and HC
C20	M	7	2	13.69	5	1.13	NR	NR
C21	M	8	5	19.41	5	4.57	NR	MC and HC
C22	F	6	4	31.56	5	1.25	NR	NR
C23	M	9	4	25.34	5	0.41	NR	NR
C24	F	13	5	242.99	7	0.74	NR	MC and HC
C25	M	6	3	21.84	5	1.16	NR	MC and HC
C26	M	8	4	20.08	5	8.98	NR	NR
C27	M	13	5	95.48	7	0.59	NR	NR
C28	M	6	3	18.65	5	1.17	NR	MC and HC
C29	M	14	5	17.05	5	2.43	NR	NR
C30	F	13	5	15.52	5	0.88	NR	MC and HC
C31	M	7	5	44.09	5	1.15	NR	MC and HC
C32	M	7	2	13.43	5	2.67	NR	NR
C33	M	8	3	17.97	5	1.65	NR	NR
C34	M	9	3	16.12	5	1.39	NR	NR

C, case; CBC, complete blood count; F, female; GFR, glomerular filtration rate; HC, hypochromia; LFTs, liver function tests; M, male; MC, microcytosis; NR, normal range.

**Table 2.** Comparison Analysis Between Groups with Blood Mercury Level

Groups		% (n)	Mean	SD	*P*
Gender	Male	58.8 (20)	29.23	26.04	.101
	Female	41.2 (14)	71.47	87.59	
Place of contamination	Classroom where mercury spilled	29.4 (10)	104.23	92.76	**.021**
	Other classes	70.6 (24)	22.62	11.18	
Interaction time	1 day	20.6 (7)	15.35	3.68	**.001**
	2 day	20.6 (7)	18.23	3.86	
	3 day	11.8 (4)	25.28	4.75	
	4 day	47.1 (16)	78.06	80.39	
Hand contact	Yes	76.5 (26)	53.75	69.33	**.04**
	No	23.5 (8)	23.46	14.87	
Sniffing up close	Yes	73.5 (25)	55.29	70.31	.035
	No	23.5 (9)	22.54	14.18	
Clinical findings, complaints	No	17.6 (6)	18.98	11.21	**.018**
	Yes	82.4 (28)	52.55	66.98	
Skin findings	No	67.6 (23)	36.89	53.51	.19
	Yes	32.4 (11)	66.99	75.88	
Gastrointestinal findings	No	38.2 (13)	60.51	71.85	.312
	Yes	61.8 (21)	38.02	55.36	
Neurological findings	No	64.7 (22)	24.95	18.78	**.04**
	Yes	35.3 (12)	86.39	90.76	
Hospitalization	Intensive care	20.6 (7)	142.16	85.51	**.01**
	Pediatric clinic	79.4 (27)	21.86	10.75	
Duration of treatment	5 days	79.4 (27)	21.86	10.75	**.01**
	7 days	20.6 (7)	142.19	85.51	

Other classes: Students take mercury from the main classroom where it is spilled and transport it to other classes.

Treatment: DMPS (2,3-dimercaptopropane-1-sulfonic acid).

Skin findings: Rash, itching, redness.

Gastrointestinal findings: Abdominal pain, nausea, vomiting.

Neurological findings: Headache, vertigo, and lethargy.

**Table 3.** Blood Mercury Levels After 5 and 7 Days of Treatment and 5 Years After Treatment

Groups		5 Days Treatment	7 Days Treatment	*P*
Treatment applied after the incident	% (n)	79.4 (27)	20.6 (7)	**.01**
	Mean	21.85	142.15	
	SD	10.75	85.51	
Situation 5 years after treatment	% (n)	79.4 (27)	20.6 (7)	**.004**
	Mean	1.98	0.83	
	SD	1.87	0.18	

Treatment: DMPS (2,3-dimercaptopropane-1-sulfonic acid).

## Data Availability

The data that support the findings of this study are available on request from the corresponding author.
